# Dentists’ Opinions in Providing Oral Healthcare to Elderly People: A Questionnaire-Based Online Cross-Sectional Survey

**DOI:** 10.3390/ijerph18063257

**Published:** 2021-03-22

**Authors:** Daria Madunic, Lidia Gavic, Ivan Kovacic, Neven Vidovic, Jasen Vladislavic, Antonija Tadin

**Affiliations:** 1 Study of Dental Medicine, School of Medicine, University of Split, 21000 Split, Croatia; dariamad1995@gmail.com (D.M.); lgavic@mefst.hr (L.G.); neven.vidovic2@st.t-com.hr (N.V.); 2 Department of Prosthodontics, Study of Dental Medicine, School of medicine, University of Split School of Medicine, 21000 Split, Croatia; ikovacic@mefst.hr; 3 Department of Pulmonology, University Hospital Center Split, 21000 Split, Croatia; jvladislavic4@gmail.com; 4 Department of Restorative Dental Medicine and Endodontics, Study of Dental Medicine, School of Medicine, University of Split, 21000 Split, Croatia

**Keywords:** dentists, attitudes, elderly, geriatric dentistry

## Abstract

This cross-sectional study aimed to assess the factors in dentists’ opinions related to oral health and the treatment management of the elderly. An online questionnaire-based survey was conducted among the dentist population (*n* = 463). Respondents were divided depending on whether they attended the geriatric dentistry course during their education, and 15 questions on the Likert scale demonstrated the difference in their attitudes. The majority of respondents (61.9%) agree that dental studies should pay more attention to acquiring sufficient knowledge and skills in the treatment of the elderly, and 56.2% would like to attend a course on that subject. Compared to those who participated in the geriatric dentistry course, those who did not consider providing oral healthcare to older people find it more difficult because of its complexity and practical obstacles (37.3% vs. 54%, *p* ≤ 0.001). From the results of this study, it can be concluded that there are differences in opinion about the provision of oral healthcare to the elderly between dentists who have and who have not attended a geriatric dentist course during their education. During the dentist’s education, geriatric dentistry courses should have a significant role in providing knowledge for working with the elderly population.

## 1. Introduction

In recent years, rapid growth in the number and percentage of older people in the total population is noted worldwide, more than at any other time in human history [1]. According to the World Health Organization definition, the elderly are considered people over 65 years of age. It is predicted that people aged 60 and over will make up half of the population by 2050 [2,3]. Although increasing the global community of older people is a medical achievement, it also poses significant healthcare system challenges. The ageing society brings many issues and challenges, including maintaining oral health [4,5].

All organs are susceptible to ageing and the changes that accompany it, including the oral cavity. Good oral health can significantly contribute to the quality of daily life [6]. According to numerous studies conducted in different countries, the oral health of the elderly is flawed, and this results in a large number of lost teeth, dental caries, frequent periodontal diseases, decreased salivary gland function, and oral precancerous/cancerous conditions [6,7,8,9]. Oral health is primarily influenced by nutrition, genetics, and general health and hygiene, and with age, these factors only grow in influence. Poor oral health and dental pain can induce the reduction of eating ability, weight loss, speech impediments, problems with hydration, and decreased perceptions of personal appearance, which consequently lead to a loss of interest in social interactions [6,9,10,11]. Oral health is an essential element of the overall health and quality of life in an individual’s life, which is why its continued promotion is necessary [12]. The demand for dental interventions among this population is much lower than is truly needed. The elderly can experience numerous barriers when going to the dentist, while dentists can experience difficulties in treating them. Barriers to dental healthcare for the elderly make access to the dentist’s office complex, especially for those living in rural areas with low public transport. Furthermore, poor post-retirement socioeconomic status (high treatment costs), coupled with a lack of habit of going to the dentist, lack of oral health knowledge, and dental fear, can deter the elderly from visiting the dentist. Lack of empathy from dental staff and poor communication contribute to this [13,14,15]. Within the elderly population, functionally dependent people placed in nursing homes are at increased risk for developing an oral illness. The lack of interest in treating these individuals is often due to the facility’s distance, lack of dentists’ time, no financial benefit, and poor education of the staff working in nursing homes [16].

Rare studies have shown slightly hostile or indifferent standpoints of dentists to treat the elderly and a low level of knowledge about the ageing process and the needs of the elderly [17,18]. Similar results were conducted among dental students [19,20]. Dentists’ views on the oral health of the elderly do not depend on the amount of knowledge on the subject, but on the frequency of encountering and treating these patients. However, the contrary is often assumed. As most seniors use public health services, dentists working in these sectors have more experience and knowledge about treating them [21]. From their own experience, dentists generally believe that the health of the elderly is inadequate, and that more should be done to motivate them to take better care of their oral health. Furthermore, numerous difficulties prevent them from adequately treating the elderly, especially the disabled. Most often, these are a lack of time and insufficient financial compensation [15,22]. Important mechanisms for improving the oral health of the elderly are based on raising awareness of the importance of oral health, encouraging preventative programs at the national level within the framework of dental healthcare, and educating and training staff to provide services and care for the elderly [23,24].

In 2009, the European College of Gerodontology published the undergraduate curriculum guidelines in gerodontology to introduce this course in dental schools throughout Europe [25,26]. Geriatric dentistry has been established in the curricula of undergraduate dentistry courses in Croatia since 1994. All four Croatian dental schools have a particular geriatric dentistry education module integrated into the undergraduate curriculum during the fifth or sixth year of the program as an independent course, followed by the lectures in other courses. In three dental schools, it is mandatory, while in one, it is an elective/optional course. This course is taught by a multidisciplinary team from different departments (endodontics, restorative dentistry, prosthodontics, oral surgery, oral medicine, and periodontology) that provides curriculum content thought didactic lectures and seminars. Only one school has clinical components in a geriatric dentistry course. The topics underlying the geriatric dental program include a wide range of issues that can be classified as traditional, with the majority focusing on geriatric medical and oral problems, diagnosis and management and oral manifestations of systemic disease in the ageing, geriatric nutrition, oral mucosal diseases of older people, prosthodontics, and periodontal, endodontic, restorative, and surgical treatment of the older patient.

This study aimed to evaluate dentists’ opinions about attitudes, knowledge, and barriers in providing oral health services to older people. The specific aim was to determine the difference between these viewpoints on deepening their undergraduate geriatric dental education. The null hypothesis was that there would be no differences in dentists’ opinions, depending on whether they attended a geriatric dentistry course during their dental education.

## 2. Materials and Methods

### 2.1. Study Design and Ethical Approval

The online, email-based, cross-sectional study was designed to assess dentists’ opinions regarding their work with the elderly. It was conducted at the Department of Restorative Dental Medicine and Endodontics, Study of Dental Medicine, School of Medicine, the University of Split during March 2019. The study was approved by the Ethics Committee of the School of Medicine, the University of Split (No: 2181-198-03-04-19-0057), who also confirmed that the research was in full accordance with ethical principles, including the World Medical Association Declaration of Helsinki (version 2013). The study was performed following the Reporting of Observational Studies in Epidemiology (STROBE) guidelines. Information about the study was included in the email to the potential participants, stating that participation was voluntary, anonymous, and without compensation. Informed consent was considered to be obtained through the act of responding.

### 2.2. Study Population

The study was conducted among dentists who had at least one year of working experience at a dental office. Only dentists in active clinical work were included in the sample, and those living and working in Croatia. The exclusion criteria were participants aged ≥ 65 years and those who had provided incomplete responses in the questionnaire.

The minimum required sample size (*n* = 358) was calculated from the total number of active members of the Croatian Chamber of Dental Medicine in 2019 (*n* = 5114) with a 95% confidence interval, a 5% margin of error, and a population proportion of 50%.

The online questionnaire designed in Google Forms was sent to 700 randomly generated dentists’ email addresses. The total response rate was 463 (66.1%). The majority of the participants were females (68.3%). The mean age of all respondents was 39.7 ± 10.7. Furthermore, the mean age of respondents who attended geriatric dentistry courses during their undergraduate education was 33.6 ± 6.6, while, for those who did not, it was 45.7 ± 9.9.

#### Measurement

A self-administered questionnaire on the dentists’ opinions in providing oral healthcare to the elderly was developed based on a relevant literature review [27].

The main questionnaire was arranged into three sections. The first section consisted of questions related to participant demographics and professional data (age, gender, year of graduation, academic degree, workplace, type of practice, number of working hours per day, number of patients per working day, and the proportion of elderly patients in the total number of patients). The second section consisted of six questions addressing the oral health opinions of elderly patients. The third section involved 15 items regarding respondents’ views on knowledge, attitudes, and barriers in providing oral care to older people, employing a Likert scale. Dentists’ opinions were rated on a five-category Likert-type scale (1 = totally disagree, 5 = totally agree). For the sake of simplicity, results were divided into three groups: 0—disagree (1 and 2), 1—neither agree nor disagree (3), 2—agree (4 and 5).

The questionnaire was initially designed in English and translated into Croatian by experts in the field [27]. After translation and back translation, to assure the quality of the data, the questionnaire was pilot tested on 25 dentists who were not included among the study participants. These participants determined the acceptability and clarity of the questions to confirm their face validity. The reliability of the opinion questionnaires was checked, and the value of Cronbach’s alpha was 0.793.

Primary outcomes were dentists’ opinions regarding knowledge, attitudes, and barriers to providing oral healthcare to the elderly. The attendance of geriatric dentistry courses during undergraduate education was considered an independent variable in the analysis, and the whole sample was divided into two groups according to that. The secondary outcome was respondents’ views on knowledge, attitudes, and barriers in providing oral care to older people, depending on demographic and personal characteristics.

### 2.3. Statistical Analysis

Data were analyzed by the Statistical Package for the Social Sciences version 25 (SPSS, IBM Corp, Armonk, New York, NY, USA). The participants were grouped depending on their education in the field of geriatric dentistry. Categorical variables were presented as whole numbers (*n*) and percentages (%), while means and standard deviations were used for quantitative variables. Statistical analyses were conducted using the Pearson’s chi-square, Fisher’s, and the Mann–Whitney U tests. Multiple linear regression analysis was used to determine the relationship of the dentists’ opinions about providing oral healthcare to the elderly and their personal and professional data. The level of statistical significance was set at *p* < 0.05.

## 3. Results

The respondents’ personal and professional data are presented in Table 1. The total number of respondents who participated in this survey was 463, while two-thirds (68.3%) of the respondents were female. Half of the sample (*n* = 229, 49.5%) had 10 to 15 patients per day, and 42.1% had 11–30% elderly (≥65 years) patients in the total number of patients. The ratio between the respondents who attended geriatric dentistry courses during their education versus those who did not attend was 49.2% versus 50.7%. More than half (56.2%) of the respondents indicated that they would like to participate in a course on oral medicine related to oral health and oral care in the elderly. Depending on whether or not they had participated in geriatric dentistry courses during their education, and considering personal and professional data, a statistically significant difference was observed related to gender (*p* ≤ 0.001), age (*p* ≤ 0.001), year of graduation (*p* ≤ 0.001), number of working hours (*p* ≤ 0.001), qualification (*p* = 0.004), and workplace (*p* = 0.002).

Table 2 shows the opinions of the respondents related to the oral health of the elderly, depending on whether they attended a geriatric dentistry course or not. Most of the respondents believed that older adults have poor oral health and do not regularly attend dental examinations (54.2% and 66.9%, respectively). Furthermore, 60% of dentists (*n* = 278) did not think that the elderly should come to regular check-ups more often than the rest of the population. Over 80% (*n* = 386) considered poor oral health a risk factor for general health, and 70% (*n* = 325) believed that tooth loss is not necessarily an ageing consequence.

Table 3 presents the respondents’ opinions about attitudes, knowledge, and barriers in providing oral health services to older people, depending on whether they attended a geriatric dentistry course during their studies. Those who did not participate in a course during their study felt that more attention should be paid to sufficient knowledge of treating ageing people in dental schools instead of those who have attended a course (166 vs. 121, *p* ≤ 0.001). Also, more of those participants stated that work with the elderly is difficult because of its complexity and practical obstacles (127 vs. 85, *p* ≤ 0.001); moreover, they considered insufficient healthcare reimbursement for dental care of the elderly as a barrier for a professional commitment compared to those who participated in a course (99 vs. 72, *p* ≤ 0.0001). Likewise, those who had participated in a course considered themselves able to provide oral healthcare to cognitively impaired older adults more than those who have not attended one (171 vs. 153, *p* = 0.028).

The results shown in Table 4 indicate that the attendance of geriatric dentistry courses during undergraduate education is a statistically significant predictor of respondents’ knowledge regarding (adverse) effects of medicines commonly used by older people (β = 0.265, SE = 0.91, *p* = 0.004), and willingness to do a regular dental examination to an elderly and infirm person via a home visit (β = 0.170, SE = 0.070, *p* = 0.015). Furthermore, the attendance of geriatric dentistry courses during undergraduate education is negatively correlated with the opinion that providing oral healthcare to older people is difficult because of its complexity and practical obstacles (β = −0.294, SE = 0.94, *p* = 0.002), and that insufficient reimbursement for the provision of oral healthcare to older people is a barrier to the professional (β = −0.208, SE = 0.94, *p* = 0.027). A detailed analysis of the regression models is shown in Table 4.

## 4. Discussion

The aim of this study was to assess dentists’ attitudes, experience, and practice towards oral healthcare of the elderly. Four hundred and sixty-three dentists answered surveys, which is 9.1% of the total number of dentists in Croatia. A similar number of respondents attended (*n* = 228) and did not attend (*n* = 235) geriatric dentistry courses during their education. The null hypothesis was not confirmed, since there are verified differences in attitudes, knowledge, and practice among these two groups. Respondents who have attended the course consider that providing dental care to the elderly was more demanding than to the younger patients (*p* = 0.002), and that tooth loss in the elderly is not an inevitable consequence of ageing (*p* ≤ 0.001). Poor oral health in the elderly is primarily seen with high tooth loss levels, dental caries, the prevalence of periodontal disease, xerostomia, precancerous lesions, and oral cancer. The basis for prevention relates to the detection of these diseases and conditions of the oral cavity at the earliest possible stages, which requires regular sessions with the patient. A dentist should work to discard the premise that any oral disease is an inevitable consequence of ageing [10].

The results also showed that 50.9% the geriatric dentistry course participants have sufficient knowledge about the harmful effects of medicines used by the elderly. Overall, most of the respondents (61.9%) believed that more attention should be given to geriatric patients. The same result was obtained by a Dutch study conducted among a population of dentists [27].

More than half (59.6%) respondents who attended a geriatric dentistry course considered that the possibility for a referral of older people with complicated oral health problems to a specialist is limited. While three quarters (75%) of thembelieve that their offices are easily attainable to the elderly. Furthermore, only 26.8% find that providing oral healthcare to older people is not so difficult due to its complexity and practical obstacles, and only 10.5%consider how compensation for delivering oral healthcare to vulnerable older people sufficient. About 40% of the respondents believe that insufficient reimbursement for providing oral healthcare to the elderly hinders professional commitment. The same was confirmed in two studies conducted in the Netherlands in 2016 and 2017 [27,28]. Dentists who have a higher proportion of elderly people in the total number of patients believed that physical, psychological, and social aspects may influence decision-making considering oral healthcare for older people (*p* = 0.026), and can provide oral healthcare to cognitively impaired people (*p* = 0.007). They also think that more attention is needed to acquire additional knowledge in geriatric dentistry during studies (*p* = 0.008).

Furthermore, Dutch colleagues who had a higher number of older adults among their patients proved to be more capable of treating this population [27]. An American survey from 2012 emphasizes the importance of clinical work with medically disabled elderly adults during college [29]. Unlike the Dutch study, where many dentists stated that they did not know the adverse effects of medicines used by the elderly, we did not find any difference in the distribution of responses depending on the respondents’ age or graduation year [28].

A minimal difference in attitudes was observed among respondents depending on age-related questions of adequate education during studying (*p* = 0.010). Moreira et al. [21], in their research, found no difference in knowledge on ageing and aged people among dentists depending on the dentists’ age. The same was confirmed for researchers from Brazil [30]. In this study, a minimal difference was also observed in gender-based attitudes. This premise has been confirmed in numerous similar studies [17,31]. Developing optimistic opinions towards the elderly can be vital to establish positive professional characteristics [32]. In this study, dentists with a higher proportion of the elderly in the total number of patients showed the most favorable opinions. It has already been demonstrated earlier that positive beliefs are influenced by personal experience and cultural and social values. While studying, the experience of working with professors who show empathy for the elderly influences the formation of student attitudes toward this particularly vulnerable part of the population [17,33,34]. Nochajski et al. [32] noticed a change in dental students’ attitudes concerning older people after meetings with this population in the clinic sessions. It showed that contacts in the clinic were associated with more positive attitudes. On the contrary, a study conducted in Belgium demonstrated that interactions between institutionalized older people and dental students have no influence on recently graduated dentists’ attitudes towards the institutionalized elderly [17].

This study has a few limitations. Since this was an observational, cross-sectional survey, participation was based on availability and willingness. Furthermore, dentists who chose to participate in the survey may be more interested or concerned with the elderly than those who did not participate.

## 5. Conclusions

In this study, dentists who took a geriatric dentistry course during their studies showed more positive attitudes towards working with elderly adults. Students’ contact with older patients appears to be a key element in developing a positive approach to treating individuals in this rapidly growing segment of society. Therefore, it would be advisable and desirable to introduce a compulsory subject of geriatric dentistry at every dental school so that future doctors can acquire the knowledge and skills necessary to work with disabled and cognitively handicapped older people. Professors should pay more attention to this sensitive topic, as they are mainly responsible for educating students and developing their positive attitudes in the treatment of older people. The critical solution is to promote ongoing education and training based on clinical leadership.

## Figures and Tables

**Table 1:** Personal and professional data of the respondents.

Characteristic		Total (*n* = 463)	NGC (*n* = 235)	GC (*n* = 228)	*p*
Gender	Male, *n* (%)	147 (31.7)	74 (31.5)	73 (32.0)	≤0.001 *
	Female, *n* (%)	316 (68.3)	161 (68.5)	155 (68.0)	
Mean age (SD)		39.7(10.7)	45.7(9.9)	33.6(7.5)	≤0.001 ^#^
Academic Degree	Dentist, *n* (%)	372 (80.3)	180 (76.5)	192 (84.2)	0.004 *
	Master of Science, *n* (%)	40 (8.7)	18 (7.7)	22 (9.6)	
	Doctor of Science, *n* (%)	51 (11.0)	37 (15.7)	14 (6.1)	
Workplace (region)	Northern Croatia, *n* (%)	172 (37.1)	100 (42.6)	72 (31.6)	0.002 *
	Eastern Croatia, *n* (%)	58 (12.5)	22 (9.4)	36 (15.8)	
	Southern Croatia, *n* (%)	187 (40.4)	83 (35.3)	104 (45.6)	
	Western Croatia, *n* (%)	46 (9.9)	30 (12.8)	16 (7.0)	
Type of practice (specialty)	General dental practitioner, *n* (%)	402 (86.8)	195 (83.0)	206 (90.4)	0.014 ^#^
	Specialist, *n* (%)	61 (13.2)	40 (17.0)	22 (9.6)	
Year of dental graduation	1979 or earlier, *n* (%)	11 (2.4)	10 (4.3)	1 (0.4)	≤0.001 **
	1980–1989, *n* (%)	57 (12.3)	53 (22.6)	4(1.8)	
	1990–1999, *n* (%)	101 (21.8)	91 (38.7)	10 (4.4)	
	2000–2010, *n* (%)	161 (34.8)	60 (25.5)	101 (44.3)	
	2010 or later, *n* (%)	133 (28.7)	21 (8.9)	112 (49.1)	
Number of working hours per day	≤8, *n* (%)	340 (73.4)	190 (80.8)	150 (65.7)	≤0.001 *
	>8, *n* (%)	123 (26.6)	45 (19.1)	78 (34.2)	
Number of patients per working day	<10, *n* (%)	128 (27.6)	68 (28.9)	60 (26.3)	0.267 *
	10–15, *n* (%)	229 (49.5)	121 (51.5)	108 (47.4)	
	>15, *n* (%)	106 (22.9)	46 (19.6)	60 (26.3)	
The proportion of elderly patients (≥65 years) in the total number of patients	≤10%, *n* (%)	94 (20.3)	52 (22.1)	42 (18.4)	0.321 *
	11–30%, *n* (%)	195 (42.1)	103 (43.8)	92 (40.4)	
	>31%, *n* (%)	174 (37.6)	80 (34.0)	94 (41.2)	
Desire to attend a course/congress on the subject of geriatric dentistry	No, *n* (%)	203 (43.8)	111 (47.2)	92 (40.4)	0.081 *
	Yes, *n* (%)	260 (56.2)	124 (52.8)	136 (59.6)	

Data are shown as the mean (standard deviation) or as a number and percentage. Statistical significance was tested by the * chi-square, ** Fisher’s, or ^#^ Mann–Whitney U tests. Statistical significance was set to *p* < 0.05. Abbreviations: NGC—did not attend a geriatric dentistry course; GC—attended a geriatric dentistry course; SD—standard deviation.

**Table 2:** Respondents’ opinion on the oral health of the elderly (*n* = 463).

Opinion		NGC (*n* = 235)	GC (*n* = 228)	*p*
Oral health of the elderly in Croatia	Bad	132 (56.1)	119 (52.2)	0.020
	Sufficient	85 (36.2)	79 (34.6)	
	Good	18 (7.7)	30 (13.2)	
Older people regularly come to dental examinations	(Totally/partially) disagree	179 (76.2)	131 (57.5)	0.053
	Neither agree nor disagree	16 (6.8)	21 (9.2)	
	(Totally/partially) agree	40 (17.0)	76 (33.3)	
Older people should come to the dental exam more often than younger people	(Totally/partially) disagree	142 (60.4)	136 (59.6)	0.980
	Neither agree nor disagree	74 (31.5)	75 (32.9)	
	(Totally/partially) agree	19 (8.1)	17 (7.5)	
Providing dental care to older people is more demanding than it is to younger patients	(Totally/partially) disagree	138 (58.7)	109 (47.8)	0.002
	Neither agree nor disagree	1 (0.4)	13 (5.7)	
	(Totally/partially) agree	96 (40.9)	106 (46.5)	
In the elderly, poor oral health is considered a risk factor for general health problems	(Totally/partially) disagree	9 (3.8)	9 (3.9)	0.736
	Neither agree nor disagree	32 (13.6)	27 (11.8)	
	(Totally/partially) agree	194 (82.6)	192 (84.2)	
Tooth loss in the elderly is an inevitable consequence of aging	(Totally/partially) disagree	149 (63.4)	176 (77.2)	≤0.001
	Neither agree nor disagree	43 (18.3)	29 (12.7)	
	(Totally/partially) agree	43 (18.3)	23 (10.1)	

Data are shown as a number and percentage. Statistical significance was tested by the chi-squared test, df = 2. Statistical significance was set to *p* < 0.05. Abbreviations: NGC—did not attend a geriatric dentistry course; GC—attended a geriatric dentistry course.

**Table 3:** Respondents’ opinions on knowledge, attitudes, and barriers in providing oral care to older people depending on whether or not they attended a geriatric dentistry course during their education.

Question		NGC (*n* = 235)	GC (*n* = 228)	*p*
Q1—Physical, psychological, and social aspects may influence decision-making considering oral healthcare for older people	(Totally/partially) agree	162 (68.9)	156 (68.4)	0.020 *
	Neither agree nor disagree	34 (14.5)	50 (29.1)	
	(Totally/partially) disagree	39 (16.6)	22 (9.6)	
Q2—I have sufficient knowledge of the (adverse) effects of medicines commonly used by older people	(Totally/partially) agree	97 (41.3)	116 (50.9)	0.053 *
	Neither agree nor disagree	74 (31.5)	69 (30.3)	
	(Totally/partially) disagree	64 (27.2)	43 (18.9)	
Q3—I am able to provide oral healthcare to cognitively impaired seniors	(Totally/partially) agree	149 (63.4)	139 (61.0)	0.463 *
	Neither agree nor disagree	69 (29.4)	77 (33.8)	
	(Totally/partially) disagree	17 (7.2)	12 (5.3)	
Q4—Dental medicine studies should pay more attention to the acquisition of sufficient knowledge and skills in the treatment of older people	(Totally/partially) agree	166 (70.6)	121 (53.0)	≤0.001 *
	Neither agree nor disagree	60 (25.5)	91 (39.9)	
	(Totally/partially) disagree	9 (3.83)	16 (7.0)	
Q5—Oral hygiene is a prerequisite for preventing oral health problems in older people	(Totally/partially) agree	223 (94.8)	211 (92.5)	0.542 **
	Neither agree nor disagree	10 (4.3)	15 (6.6)	
	(Totally/partially) disagree	2 (0.9)	2 (0.9)	
Q6—Each dentist is responsible for providing proper oral healthcare to elderly people who are unable to leave their home, but who have previously regularly come to their practice (with the precondition that they are their patients)	(Totally/partially) agree	117 (49.8)	119 (52.2)	0.051 *
	Neither agree nor disagree	70 (29.8)	81 (35.5)	
	(Totally/partially) disagree	48 (20.4)	28 (12.3)	
Q7—I am prepared to do a regular dental examination to an elderly and infirm person via a home visit	(Totally/partially) agree	162 (68.9)	174 (76.3)	0.182 *
	Neither agree nor disagree	54 (23.0)	42 (18.4)	
	(Totally/partially) disagree	19 (8.1)	12 (5.3)	
Q8—I have repeatedly experienced that at some point older, disabled people stopped coming for regular check-ups (appointments)	(Totally/partially) agree	142 (60.4)	139 (60.9)	0.007 *
	Neither agree nor disagree	48 (20.4)	66 (28.9)	
	(Totally/partially) disagree	45 (19.1)	23 (10.2)	
Q9—From the dentist’s point of view, treating the vulnerable elderly is not too demanding	(Totally/partially) agree	43 (18.3)	44 (19.3)	0.873 *
	Neither agree nor disagree	75 (31.9)	76 (33.3)	
	(Totally/partially) disagree	117 (49.8)	108 (47.4)	
Q10—Possibilities for referrals of elderly people with complex oral health problems to fellow specialists are limited	(Totally/partially) agree	123 (52.3)	136 (59.6)	0.127 *
	Neither agree nor disagree	52 (22.1)	51 (22.4)	
	(Totally/partially) disagree	60 (25.5)	41 (18.0)	
Q11—Providing oral healthcare to older people is difficult because of its complexity and practical obstacles	(Totally/partially) agree	127 (54.0)	85 (37.3)	≤0.001 *
	Neither agree nor disagree	56 (23.8)	82 (36.0)	
	(Totally/partially) disagree	52 (22.1)	61 (26.8)	
Q12—The reimbursement for providing oral health care to vulnerable older people is insufficient	(Totally/partially) agree	148 (63.0)	121 (53.1)	0.065 *
	Neither agree nor disagree	72 (30.6)	83 (36.4)	
	(Totally/partially) disagree	15 (6.4)	24 (10.5)	
Q13—The institution (dental office) where I practice is easily accessible to the elderly (no major obstacles)	(Totally/partially) agree	153 (65.1)	171 (75.0)	0.028 *
	Neither agree nor disagree	24 (10.2)	23 (10.1)	
	(Totally/partially) disagree	58 (24.7)	34 (14.9)	
Q14—Usually, the provision of oral healthcare to the elderly involves various technical limitations	(Totally/partially) agree	121 (51.5)	103 (45.2)	0.027 *
	Neither agree nor disagree	55 (23.4)	79 (34.6)	
	(Totally/partially) disagree	59 (25.1)	46 (20.2)	
Q15—I find that insufficient reimbursement for the provision of oral healthcare to older people is a barrier to the professional commitment to this particular group of patients	(Totally/partially) agree	99 (42.1)	72 (31.6)	≤0.001 *
	Neither agree nor disagree	55 (23.4)	96 (42.1)	
	(Totally/partially) disagree	81 (34.5)	60 (26.3)	

Data are shown as a number and percentage. Statistical significance was tested by the * chi-square or ** Fisher’s tests. Statistical significance was set to *p* < 0.05. Abbreviations: NGC—did not attend a geriatric dentistry course; GC—attended a geriatric dentistry course.

**Table 4:** Multiple linear regression analysis results for respondents’ opinions on knowledge, attitudes, and barriers in providing oral care to older people, depending on their personal and professional data.

Question		Predictors					
		Gender	Age	Year of Graduation	Specialty	Number of Elderly Patients in Care	Attended Geriatric Dentistry Course
Q1—Physical, psychological, and social aspects may influence decision-making considering oral healthcare for older people	β	−0.081	−0.002	0.028	0.060	0.083	0.039
	t	0.071	0.051	0.062	0.025	0.037	0.083
	*p*	0.253	0.975	0.650	0.016	0.026	0.645
Q2—I have sufficient knowledge of the (adverse) effects of medicines commonly used by older people	β	−0.121	0.030	−0.052	0.027	0.060	0.265
	t	0.077	0.055	0.067	0.027	0.040	0.091
	*p*	0.118	0.586	0.436	0.321	0.135	0.004
Q3—I am able to provide oral healthcare to cognitively impaired seniors	β	−0.078	0.101	0.099	0.041	0.085	−0.005
	t	0.060	0.043	0.052	0.021	0.031	0.070
	*p*	0.193	0.018	0.057	0.048	0.007	0.939
Q4—Dental medicine studies should pay more attention to the acquisition of sufficient knowledge and skills in the treatment of older people	β	0.049	0.108	0.113	0.012	0.081	−0.209
	t	0.058	0.042	0.050	0.020	0.030	0.068
	*p*	0.401	0.010	0.025	0.555	0.008	0.002
Q5—Oral hygiene is a prerequisite for preventing oral health problems in older people	β	−0.035	0.011	−0.010	0.001	0.006	0.005
	t	0.029	0.021	0.025	0.010	0.015	0.034
	*p*	0.233	0.595	0.696	0.939	0.679	0.886
Q6—Each dentist is responsible for providing proper oral healthcare to elderly people who are unable to leave their home, but who have previously regularly come to their practice (with the precondition that they are their patients)	β	−0.036	0.102	0.124	0.100	0.131	0.101
	t	0.072	0.052	0.063	0.025	0.038	0.085
	*p*	0.615	0.049	0.049	≤0.001	≤0.001	0.239
Q7—I am prepared to do a regular dental examination to an elderly and infirm person via a home visit	β	−0.070	0.067	0.022	0.036	−0.012	0.170
	t	0.059	0.043	0.052	0.021	0.031	0.070
	*p*	0.238	0.114	0.667	0.084	0.708	0.015
Q8—I have repeatedly experienced that at some point older, disabled people stopped coming for regular check-ups (appointments)	β	0.020	−0.010	−0.001	−0.065	0.153	0.026
	t	0.071	0.051	0.062	0.025	0.037	0.084
	*p*	0.776	0.843	0.992	0.009	≤0.001	0.753
Q9—From the dentist’s point of view, treating the vulnerable elderly is not too demanding	β	−0.006	0.066	−0.007	0.017	0.056	0.126
	t	0.077	0.055	0.067	0.027	0.040	0.090
	*p*	0.939	0.228	0.913	0.531	0.164	0.164
Q10—Possibilities for referrals of elderly people with complex oral health problems to fellow specialists are limited	β	0.265	0.091	0.141	−0.027	−0.020	0.102
	t	0.079	0.057	0.069	0.028	0.042	0.093
	*p*	≤0.001	0.107	0.041	0.326	0.634	0.275
Q11—Providing oral healthcare to older people is difficult because of its complexity and practical obstacles	β	0.097	−0.010	0.082	0.078	−0.051	−0.294
	t	0.079	0.057	0.069	0.028	0.042	0.094
	*p*	0.225	0.856	0.237	0.005	0.221	0.002
Q12—The reimbursement for providing oral healthcare to vulnerable older people is insufficient	β	0.054	0.068	0.057	0.063	0.003	−0.098
	t	0.064	0.046	0.056	0.022	0.034	0.076
	*p*	0.400	0.139	0.306	0.005	0.925	0.195
Q13—The institution (dental office) where I practice is easily accessible to the elderly (no major obstacles)	β	−0.082	−0.045	0.010	−0.116	−0.007	0.090
	t	0.079	0.056	0.069	0.028	0.041	0.093
	*p*	0.296	0.430	0.888	≤0.001	0.871	0.334
Q14—Usually, the provision of oral healthcare to the elderly involves various technical limitations	β	0.195	0.157	0.242	0.133	−0.003	−0.058
	t	0.077	0.055	0.067	0.027	0.040	0.091
	*p*	0.012	0.005	≤0.001	≤0.001	0.947	0.522
Q15—I find that insufficient reimbursement for the provision of oral healthcare to older people is a barrier to the professional commitment to this particular group of patients	β	0.115	−0.003	0.150	0.104	0.126	−0.208
	t	0.079	0.057	0.069	0.028	0.042	0.094
	*p*	0.149	0.959	0.030	≤0.001	0.003	0.027

## Data Availability

The data that support the findings of this study are available from the corresponding author upon reasonable request.
